# Kratom as a potential substance use disorder harm reduction agent

**DOI:** 10.3389/fpubh.2024.1416689

**Published:** 2024-05-30

**Authors:** MeShell Green, Nina Vadiei, Charles A. Veltri, Oliver Grundmann, Kirk E. Evoy

**Affiliations:** ^1^Midwestern University College of Pharmacy, Glendale, AZ, United States; ^2^The University of Texas at Austin College of Pharmacy, Austin, TX, United States; ^3^San Antonio State Hospital, San Antonio, TX, United States; ^4^University of Florida College of Pharmacy, Gainesville, FL, United States; ^5^Department of Pharmacy, University Health, San Antonio, TX, United States

**Keywords:** kratom, *Mitragyna speciosa*, mitragynine, substance use disorder, alcohol use disorder, opioid use disorder, stimulant use disorder, harm reduction

## Abstract

Substance use disorders contribute to considerable U.S. morbidity and mortality. While effective pharmacotherapy options are available to treat opioid and alcohol use disorders, for a variety of reasons, many patients lack access to treatment or may be reluctant to seek care due to concerns such as perceived stigma or a current lack of desire to completely curtail their substance use. Furthermore, treatment options are limited for patients with stimulant or polysubstance use disorders. Thus, there is considerable need to expand the substance use disorder harm reduction armamentarium. Kratom (*Mitragyna speciosa* Korth.) is an herbal substance that can produce both opioid and stimulant-like effects, and its use in the US is growing. Though there are concerns regarding adverse effects, dependence risk, and limited regulation of its manufacturing and sale, the pharmacology of kratom and early preclinical studies suggest a potential role as a harm reduction agent for various substance use disorders, and it has historically been used in Southeast Asia for such purposes. The goal of this review is to describe kratom’s history of use, pharmacology, and early pre-clinical and observational research regarding its therapeutic potential in opioid use disorder, as well as alcohol, stimulant, and polysubstance use disorders, while also highlighting current concerns around its use, existing gaps in the literature, and directions for future research.

## 1 Introduction

Kratom leaves represent a natural herbal substance that can produce both opioid and stimulant-like effects (1). Increased United States (U.S.) kratom use has caused concern among clinicians regarding its risks, potential for misuse and dependence, and lacking regulation. However, a limited but growing body of literature purports kratom’s potential as a harm reduction agent to mitigate overdose risk for individuals with substance use disorders (SUD). This review describes evidence for kratom’s potential efficacy in helping to manage various SUDs and the need for additional research.

### 1.1 History and patterns of use

The tree used to produce kratom products, *Mitragyna speciosa* (Korth.,) Havil., is native to Malaysia, Indonesia, Borneo, Philippines, and New Guinea (2). Its leaves have historically been consumed by indigenous communities as a mild stimulant, an ethnomedicine to treat pain, diarrhea, fever, or mental health conditions, and as an alternative to opioids or stimulants, helping to reduce use or withdrawal symptoms (3).

U.S. kratom consumption has increased over the past two decades, with lifetime and past-year use estimates ranging from 0.5–6.1% and 0.7–4.1% of the population, respectively, with a higher prevalence among SUD populations (past-year and lifetime use: 10 and 21%, respectively) (4–9). Kratom user demographics skew toward employed White males in their late teens to middle-age adults, with at least some college education (6, 10, 11).

Consumption patterns are variable, with a wide range in frequency, dose, and administration route. Small doses tend to be consumed for mild stimulant effects or to mitigate health-related symptoms, while larger doses produce more pronounced psychoactive effects (12). This variable consumption, coupled with numerous preparations available and limited regulatory oversight, highlight both potential benefits and risks. Two areas of benefit reported by kratom users are to alleviate withdrawal symptoms and reduce opioid, stimulant, or alcohol cravings, emphasizing kratom’s potential as a harm reduction agent for various SUDs (13–16). Furthering this potential is the notion that kratom confers less overdose or dependence risk and does not produce as potent of euphoric effects as traditional opioids (17, 18).

However, several concerns regarding kratom use warrant additional research before it can be recommended in clinical practice. Though generally tolerated at low and slowly titrated doses, kratom can produce adverse effects in line with their mild opioid and stimulant effects, including nausea, somnolence, confusion, agitation, hypertension, and tachycardia that are generally mild (10, 19). Case reports describe more serious, rare adverse events, including seizures, hepatotoxicity, cardiac arrest, or overdose (13, 20, 21). It is also well-established that routine kratom use can lead to dependence and withdrawal upon abrupt discontinuation (1). Controlled clinical studies are lacking to adequately assess adverse effect prevalence, and safety information is largely based on self-reported, observational data, spontaneous reporting systems, or case reports of rare events. U.S. poison control center data indicate increasing reported incidents in recent years, likely due to greater availability and use. Among 1,807 kratom-related reports from 2011 to 2017, 65% occurred between 2016 and 2017, and a subsequent analysis identified a 90% increase from 2017 to 2018 (22, 23). In a separate study, 7.4% of reported incidents were deemed major or life-threatening (19). There is also concern that, given the lack of regulatory oversight of kratom manufacturing and sales, available products are highly variable in content, may be mislabeled regarding the concentration of active compounds, and may contain toxic metals or potentially infectious microbes (24).

### 1.2 Current regulatory state

The US Food and Drug Administration (FDA) classifies kratom products as new dietary ingredients, meaning there is currently insufficient evidence to support medical use or provide assurance that it does not pose significant health risks when consumed (25). Kratom is not scheduled under the U.S. Controlled Substances Act and federal regulations and oversight are minimal. Regulations are instead at the state level through “Kratom Consumer Protection Acts” which focus on labeling, adulteration, and age restrictions. This has led to the medley of kratom products in the U.S. market, ranging from dried leaf preparations more representative of the plant’s natural chemistry to highly concentrated extracts enriched with active alkaloids. This accentuates the need for better control of kratom-derived products and manufacturing practices and controlled clinical trials to establish safety, efficacy, and optimal dosing.

### 1.3 Pharmacology and medicinal chemistry

Kratom products contain many phytochemicals, including at least 40 distinct alkaloids (3). Mitragynine is the most abundant alkaloid (66%) and most associated with kratom’s pharmacological and clinical effects, acting as a partial agonist at the μ-opioid receptor, a weak competitive antagonist at the κ- and δ-opioid receptors, and a partial agonist at several serotonin and α-adrenergic receptors (26, 27). Importantly, mitragynine does not recruit β-arrestin-2, thereby reducing its respiratory depression risk (28). Speciocilliatine, speciogynine, paynantheine, and corynantheidine are structural isomers of mitragynine, making up 20% of the alkaloid content. These four alkaloids show greater affinity toward one of the three signaling pathways (nociceptive, serotonergic, or α-adrenergic) and weaker interactions with targets in the other two. Minor alkaloids (7-hydroxymitragynine, mitraciliatine, and isopaynantheine) also show opioid receptor-mediated antinociception. Notably, 7-hydroxymitragynine, both an oxidative degradation product of drying kratom leaves and a metabolite generated in the body, possesses 10-fold higher binding affinity than mitragynine (29). While mitragynine is only 25% as potent as morphine *in vivo*, 7-hydroxymitragynine possesses 10x times greater potency than morphine at opioid receptors. This complex mixture of active compounds and influence on signaling pathways points to kratom as a possible treatment for mitigating symptoms and withdrawal of various SUDs (30).

## 2 Opioid use disorder

In 2021, opioid overdoses caused more than 80,000 U.S. deaths (31). Many others develop communicable diseases from high-risk opioid administration. Despite availability of several FDA-approved pharmacologic agents for opioid use disorder (OUD, i.e., methadone, buprenorphine, and naltrexone), many patients lack access or do not seek assistance from healthcare providers. There remains a need to expand the harm reduction armamentarium.

Several pre-clinical studies confirmed antinociceptive effects of mitragynine and 7-hydroxymitragynine. Antinociceptive effects were determined in the hot plate test in rats using mitragynine or morphine (32). While morphine reduced operant response at low doses, mitragynine required higher doses. There is also evidence that at least some of its effects are not primarily mediated through opioid receptors (33, 34). Morphine antinociceptive effects were fully antagonized by administration of the opioid antagonist naltrexone, whereas mitragynine could not be fully antagonized. However, another rat study determined that mitragynine withdrawal can be precipitated with the opioid receptor antagonist naloxone and rimonabant, a cannabinoid receptor-1 antagonist (35). Mitragynine and buprenorphine, a partial μ-opioid receptor agonist commonly used as a medication for OUD, both ameliorated morphine-induced withdrawal. This indicates that mitragynine can prevent morphine-precipitated withdrawal, potentially through actions at the opioid and cannabinoid receptors (35). Effects on the adrenergic receptors may also contribute, though this has yet to be evaluated in controlled studies. Less pronounced and shorter lasting precipitated mitragynine withdrawal indicates lower dependence and misuse potential versus classic opioids.

To further support lower misuse liability, a conditioned place preference (CPP) model compared continued self-administration of mitragynine to morphine. Whereas rats provided with morphine increased their intake over the duration of the study, animals provided mitragynine maintained their intake and reduced morphine intake if both were co-administered (36). In addition, precipitated withdrawal using pentylenetetrazole did not produce the anticipated anxiogenic effect for mitragynine that was observed for morphine (37, 38).

An important differentiator between pre-clinical animal studies and human studies is the administration route, which is primarily intravenous or intraperitoneal for animals, thus resulting in higher absorption and decreased alkaloid metabolism (39). Oral mitragynine bioavailability is low (30%) with subsequent metabolism to 7-hydroxymitragynine, mitragynine pseudoindoxyl, and inactive metabolites (40–42). The contribution of 7-hydroxymitragynine and mitragynine pseudoindoxyl to kratom’s opioid-like effects remains unknown.

Whole kratom leaf products have not been well studied for OUD. One mouse study evaluated effects of lyophilized kratom tea containing 7.4 mg/g mitragynine, 3.5 mg/g speciociliatine, and 2.5 mg/g paynantheine (43). The tea, in oral doses of 100 and 1,000 mg/kg, produced antinociception lasting up to 60 min. Kratom tea had less pronounced, shorter-lasting respiratory depression than the morphine control, and did not present with CPP (an indicator of dependence) as morphine did. Furthermore, kratom tea administration following a four- or seven-day morphine induction significantly reduced several withdrawal behaviors, indicating that kratom tea can reduce opioid withdrawal symptoms.

One Malaysian kratom user study evaluated people with concomitant opioid and stimulant use disorders (44). Most reported using kratom to treat heroin withdrawal (77%), as a heroin replacement (59%), and to reduce heroin intake (58%), independent of how long they used kratom. Only 15% of 332 users reported consuming kratom for euphoric effects. This study supports traditional kratom use as an OUD harm reduction strategy versus using to obtain euphoric effects, though it’s important to note potential sample selection bias and generalizability concerns given the study design.

## 3 Alcohol use disorder

Alcohol use disorder (AUD) is the most common SUD in the U.S., (45) yet has very few evidence-based treatments, each with only modest efficacy in reducing alcohol consumption and improving morbidity/mortality. A 2017 online survey conducted with support from the American Kratom Association studied 3,024 current and past kratom users (46). Of those currently using kratom (*N* = 2,867), 2.2% (*N* = 64) acknowledged mainly using kratom to reduce or quit alcohol consumption, whereas 18.0% (*N* = 517) included it among multiple reasons for using kratom.

Preclinical investigations have explored kratom efficacy in therapeutic AUD management. Animal studies suggest that kratom’s delta opioid receptor (DOR) antagonistic activity may attenuate alcohol intake (47). Orally administered 50–100 mg/kg *Mitragyna speciosa* leaf extract doses reduced elevated dopamine levels in ethanol-dependent mice. Furthermore, kratom alkaloids exhibit activity at mu, delta, and kappa opioid receptors with negligible β-arrestin-2 recruitment, earning the G protein-biased partial agonist designation (48). Preclinical studies demonstrated that G protein-biased partial agonists targeting DOR reduced mouse alcohol intake, while increased β-arrestin-2 recruitment was linked to enhanced alcohol consumption (48). Another study revealed that 7-hydroxymitragynine modulates alcohol intake at the DOR in mice; however, its elevated potency at the mu opioid receptor (MOR) raises dependency, misuse, and adverse event concerns (48). Most recently, a preclinical study utilized kratom alkaloids with a lower MOR potency than 7-hydroxymitragynine (49). Wildtype mice displayed dose-dependent voluntary alcohol consumption reductions, while DOR knockout mice did not exhibit the same pattern. This corroborates previous research indicating involvement of G-protein biased agonism of DOR in AUD management.

*Mitragynine* also attenuated ethanol withdrawal symptoms in rats. Intragastric administration of 100, 300, and 500 mg/kg doses decreased immobility duration by 25% versus normal saline, without significant effect on spontaneous motor activity (50). 10 h after ethanol cessation, withdrawal-related behaviors remained suppressed. Another preclinical investigation determined that an alkaloid extract derived from *M. speciosa* reduced ethanol withdrawal symptom severity without inducing spontaneous motor activity (50).

These studies provide insights regarding kratom’s potential application for AUD treatment; nevertheless, further research is required to comprehensively evaluate its efficacy, adverse effects, and optimal human dosing, as regular use may pose more risks than existing treatment options.

## 4 Stimulant use disorder

Global concern over stimulant misuse, specifically cocaine and amphetamines, is gradually increasing. Chronic stimulant misuse is linked to elevated mortality and adverse physical and psychological outcomes, including stroke, respiratory disease, bloodborne viruses, and neurocognitive alterations (51). Presently, available interventions to address stimulant dependence offer minimal to no resolution. There are no pharmacotherapies approved for the management of dependence and withdrawal.

Several reports have indicated Kratom’s potential benefit for methamphetamine, cocaine, and polysubstance use disorder self-treatment. Among 332 Malaysian rehabilitation patients who concurrently used heroin and methamphetamine, 71% reported using kratom to reduce methamphetamine intake, while 59% used it as a methamphetamine substitute (44). More than half (59%) viewed kratom as a safer alternative to methamphetamine and heroin.

Preclinical investigations also provided insights. Kratom alkaloid extract (80 mg/kg, containing 3.8 mg mitragynine) administration significantly reversed methamphetamine CPP and suppressed nucleus accumbens (NAc) gamma I and hippocampus delta signals in mice, where addictive behavior regulation predominantly occurs (52). Local field potential (LFP) signals (which record summated excitatory and inhibitory potentials from NAc and hippocampus neurons) were observed in mice administered methamphetamine, to explore neural signaling associated with drug craving and reward-seeking behavior. LFP and locomotor activity patterns were subsequently monitored following treatment with either kratom alkaloid extract, bupropion, or control. Kratom alkaloid extract significantly ameliorated methamphetamine CPP versus other treatments. Mice conditioned with methamphetamine exhibited a significant increase in the NAc gamma I power spectra; however, 80 mg/kg kratom alkaloid extract administration significantly reversed this effect. In the hippocampus, both 40 mg/kg and 80 mg/kg kratom alkaloid extracts suppressed methamphetamine-induced delta power increases. Elevated gamma I frequency activity range is associated with dopamine release and addiction, while heightened delta rhythms may reflect stress response regulation (52). Interestingly, a positive correlation was present between delta power and locomotor activity patterns, further suggesting kratom may attenuate stimulant craving behaviors. While additional preclinical studies depicting effects of chronic kratom administration are required, this information helps explain its potential efficacy in addressing physical and mental symptoms associated with stimulant dependence and withdrawal.

## 5 Polysubstance use disorders

Concurrent opioid and stimulant use is increasingly common and has been linked to increased overdose-related deaths (53, 54). Other substances, such as alcohol, benzodiazepines, and gabapentinoids are also commonly implicated in overdose-related deaths (53, 55). Prior research suggests stimulants are commonly mixed with depressants such as opioids/alcohol/benzodiazepines to offset their negative effects, namely withdrawal symptoms (53, 56). Unfortunately, fentanyl is also increasingly present in illicit stimulants, resulting in unintentional opioid overdoses (57).

For individuals with OUD, use of additional substances to offset their ‘highs’ or minimize withdrawal symptoms suggests need for harm reduction agents that can mitigate SUD symptoms without inducing opioid withdrawal or considerably increasing overdose risk. While there are highly effective OUD treatment options available, (58) they may not be viable for individuals looking to minimize withdrawal symptoms but unready to quit their preferred substance(s). Since methadone is a full opioid receptor agonist, use in conjunction with other opioids greatly increases overdose risk. Buprenorphine (a partial opioid agonist) and naltrexone (an opioid antagonist) induce opioid withdrawal if used concurrently with full opioid agonists (59, 60). Furthermore, for a variety of reasons, many patients lack access to effective OUD treatment or may be reluctant to seek care due to concerns such as perceived stigma surrounding SUD. Kratom could have potential utility for such patients.

In a latent class analysis of U.S. kratom users, utilizing data from the 2019 National Survey on Drug Use and Health (NSDUH), 412 adults reported using kratom in the past 12-months in addition to at least one of 11 additional substances (61). The three most distinct polysubstance user profiles identified were kratom users who also were using marijuana/alcohol/tobacco [63.3%], marijuana/alcohol/tobacco + psychedelics [19.3%], and marijuana/alcohol/tobacco + psychedelics/heroin/prescriptions [17.4%]. These data suggest most U.S. kratom users are polysubstance users. They may be using to offset negative effects of multiple substances or to manage common symptoms that drive individuals to use substances (e.g., comorbid mood disorders) (62). Given that data demonstrate kratom is mostly consumed by polysubstance users, and that it may have utility for multiple SUDs, further investigation into its utility as a harm reduction tool for polysubstance use disorders is warranted.

## 6 Discussion and conclusion

This review provides insight into kratom’s pharmacological properties that indicate a possible role as a harm reduction agent for individuals with opioid, alcohol, stimulant, or polysubstance use disorders. However, much more research is needed before recommending kratom for clinical use. Future controlled clinical trials are necessary to investigate the impact of kratom in managing SUDs in human subjects, since current research is limited to heterogenous preclinical studies, case reports, and self-reported observational data (13). Furthermore, much work needs to be done to assess how to use kratom for such purposes, including studies to determine the ideal composition and optimal dose of the various chemical compounds found in kratom, the adverse effect profile and potential for drug interactions when used at these doses, and the impact of delivery route and what delivery systems would optimize its clinical effects while mitigating risks. There is also much work that needs to be done to standardize and regulate manufacturing and sale of available kratom products, to help ensure they are appropriately labeled to match the product content and are free of harmful contaminants. Despite concerns regarding misuse potential and safety, kratom use is on the rise; the development of novel kratom-derived compounds and improved regulatory standards would allow for further investigation into kratom’s potential as a harm reduction agent for individuals with SUDs, a high-risk, vulnerable population with limited treatment options.

## Author contributions

MG: Conceptualization, Writing – original draft, Writing – review & editing. NV: Conceptualization, Writing – original draft, Writing – review & editing. CV: Conceptualization, Writing – original draft, Writing – review & editing. OG: Conceptualization, Writing – original draft, Writing – review & editing. KE: Conceptualization, Writing – original draft, Writing – review & editing.
